# Novel Heat-Mitigating Chip-on-Probe for Brain Stimulation Behavior Experiments

**DOI:** 10.3390/s20247334

**Published:** 2020-12-21

**Authors:** Seongwoog Oh, Jungsuek Oh

**Affiliations:** Institute of New Media and Communications and School of Electrical and Computer Engineering, Seoul National University, Seoul 08826, Korea; dillon1859@snu.ac.kr

**Keywords:** behavior system, chip-on-probe, microwave probe, microwave monolithic integrated chip

## Abstract

This paper proposes a novel design for a chip-on-probe with the aim of overcoming the heat dissipation effect during brain stimulations using modulated microwave signals. The temperature of the stimulus chip during normal operation is generally 40 °C–60 °C, which is sufficient to cause unintended temperature effects during stimulation. This effect is particularly fatal in brain stimulation applications that require repeated stimulation. This paper proposes, for the first time, a topology that vertically separates the stimulus chip generating the stimulus signal and the probe delivering the signal into the brain to suppress the heat transfer while simultaneously minimizing the radio frequency (RF) transmission loss. As the proposed chip-on-probe should be attached to the head of a small animal, an auxiliary board with a heat sink was carefully designed considering the weight that does not affect the behavior experiment. When the transition structures are properly designed, a heat sink can be mounted to maximize the cooling effect, reducing the temperature by more than 13 °C in a simulation when the heat generated by the chip is transferred to the brain, while the transition from the chip to the probe experiences a loss of 1.2 dB. Finally, the effectiveness of the proposed design is demonstrated by fabricating a chip with the 0.28 μm silicon-on-insulator (SOI) complementary metal–oxide–semiconductor (CMOS) process and a probe with a RT6010 printed-circuit board (PCB), showing a temperature reduction of 49.8 °C with a maximum output power of 11 dBm. In the proposed chip-on-probe device, the temperature formed in the area in contact with the brain is measured at 31.1 °C.

## 1. Introduction

Brain stimulation methods such as transcranial direct current stimulation (tDCS), transcranial magnetic stimulation (TMS), and microwave brain stimulation (MBS) change or improve the function of the brain through each type of stimulus in a noninvasive manner. Over the past few decades, tDCS and TMS techniques have been studied and used to improve and treat brain disorders as representative noninvasive stimulations [1,2,3,4]. Unlike previous methods, MBS was only recently proposed as a method of brain stimulation, and it is in the relatively early stages of research [5,6,7]. Environmental changes affect the connectivity of nerves in the brain, and the extent of these effects can vary depending on the type of stimulus signal. The microwave signal used in MBS has an excellent ability to concentrate the stimulation region based on the characteristics of the short wavelengths compared to other DC currents or low-frequency magnetic fields [8].

This type of MBS system consists of a chip capable of generating a stimulus signal with a built-in modulator and a probe with a reflection coefficient of −10 dB or less. In a typical system configuration, the chip is modularized through wire bonding and then delivers the required DC voltage and RF signals, and the RF signals are applied to the probe through a coaxial cable. During the modularization process of the chip, a copper or aluminum jig is used to suppress the generated heat to operate within the temperature range to ensure performance. Considering the weight and elasticity of commonly used coaxial cables and the bulkiness of the jig, this system configuration is not suitable for a behavioral experiment because it limits the free movements of small animals. For the behavioral experiment of small animals, especially mice, the weight of the implanted device should be less than 4 g [9]. Accordingly, the use of RF cables and jigs should be avoided in the entire system.

**Figure 1:**
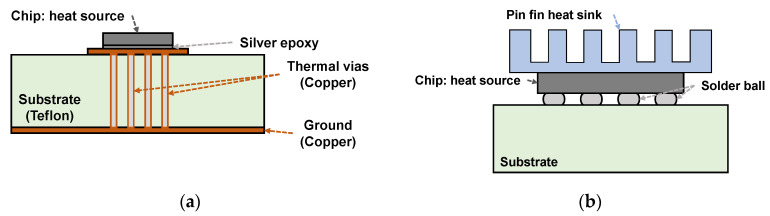
Previous methods to enhance thermal dissipation of the chip by (**a**) printed-circuit board (PCB) thermal vias and (**b**) direct heat sink attachment.

Various methods have been tried to solve the heat dissipation problem of the chip. As shown in Figure 1a, a Teflon-based thermal PCB was designed for packaging the chip to ensure efficient heat dissipation by incorporating optimized thermal vias in the design [10]. Research for work for the thermal enhancements of 2.5D is done by bridging the thermal path from the die to the top heat sink directly without molding material [11] as depicted in Figure 1b. The other work used a new photo-imageable solder resist (PSR) that has high thermal conductivity to reduce the junction temperature of the chip [12]. However, forming multiple thermal vias under and around the chip to dissipate heat efficiently is not suitable for applications that require minimum heat transfer to the underside of the PCB, which is targeted in this study. Additionally, the method in which the heat sink is directly attached to the chip die is very effective, but this is available when packaged by the relatively expensive flip-chip bonding, which is not suitable for this study implemented through wedge bonding. Using a new PSR requires an additional step in PCB fabrication, which is a method available only to limited users.

This work presents a novel design method to realize a MBS system as a chip-on-probe system considering the thermal effect and weight for utilization in animal behavior experiments. In order to suppress the heat effect transmitted to the brain tissue, the system was divided into two PCBs for the probe and for the chip and connected with RF connectors to secure the physical distance between the two PCBs. A careful transition structure was designed to minimize the transition loss from the pad of the chip to the aperture surface of the probe in contact with brain tissue. Finally, the detailed design procedure and measurement results of the proposed MBS system are described.

## 2. Design of the Chip-on-Probe System

### 2.1. Study of RF Frequency Characteristics with Brain Tissue

**Figure 2:**
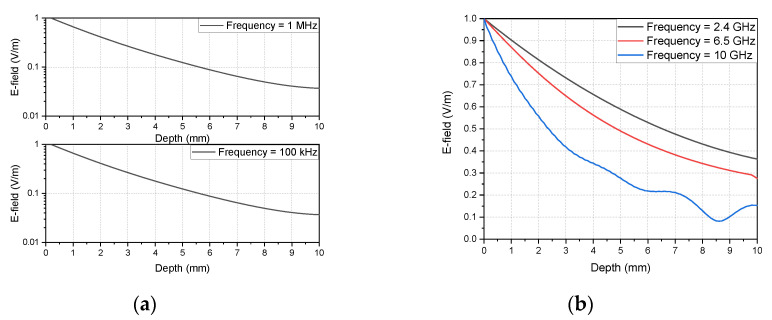
E-field attenuation in the brain tissue model for frequencies of (**a**) 100 kHz, 1 MHz, (**b**) 2.4 GHz, 6.5 GHz, and 10 GHz.

Conventional brain stimulation methods use frequencies below 1 MHz to induce changes in the brain. In this low-frequency band, the degree of attenuation does not change significantly with the frequency as the field passes through the brain. Figure 2a shows the attenuation according to the depth when an electric field enters the brain tissue. For a fair comparison, the graph is normalized to the electric field intensity at a zero depth. When the frequencies were 100 kHz and 1 MHz, respectively, they all exhibited the same attenuation rate. However, in the microwave frequency band, because the attenuation rate varies greatly with the frequency, careful consideration of the frequency is essential in the MBS, as shown in Figure 2b. To confirm the attenuation rate at microwave frequencies, changes in the electric field intensity were observed using an attenuation constant in a lossy dielectric from (1) and with CST Studio at 2.4, 6.5, and 10 GHz. The permittivity and loss tangent of the brain used for system design were obtained through the derived fourth-order Debye model [13] and utilized in the simulation. The microwave frequencies of 2.4 and 10 GHz were selected because they are unlicensed bands and high enough to show with attenuation, respectively. For the 6.5 GHz frequency, a 1/e (37%) reduction in the maximum field level of the surface is shown at a depth of 3 mm, where the hippocampus, the target site, is located. The 6.5 GHz microwave frequency has relatively low attenuation and while at the same time offers the possibility of reducing the size of the probe compared to the 10 and 2.4 GHz frequencies.

(1) $$\alpha \left(\omega \right)=\sigma \sqrt{\frac{\mu }{2\epsilon \left[1+\sqrt{1+\frac{\sigma }{\omega \epsilon }}\right]}}$$

**Figure 3:**
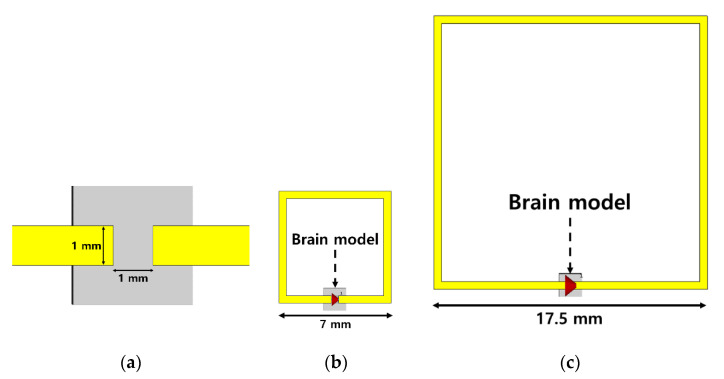
Top view of (**a**) the gap of SRR, (**b**) 2.4 GHz, and (**c**) 6.5 GHz SRR contacting the brain model.

**Figure 4:**
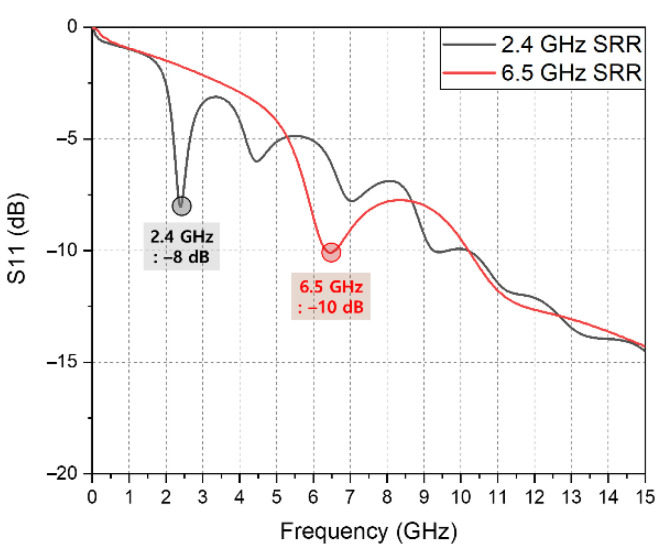
Simulated S-parameter of SRR with a brain model.

The lower the frequency, the lower the attenuation, but the larger the size of the probe which uses a resonant structure in the system. Since the application of this work is to develop a system for behavioral experiments in mice, the dimension as well as the attenuation is a limiting factor. Figure 3 shows a split ring resonator (SRR) resonating at each frequency. Given that the probe must be matched by resonance at the desired microwave frequency, a simple SRR structure can serve to predict the size of the entire probe. As mentioned above, the attenuation constant is large at 10 GHz, meaning that the size comparison is conducted in only the two frequency bands of 2.4 and 6.5 GHz. The electric field is concentrated in the gap, acting as a capacitor, and the metal line returning to the loop creates resonance as the inductance. When the mouse brain is assumed to be a sphere, the size of the area where the probe comes into contact with the brain surface is limited because the radius is smaller than 5 mm [14]. Considering the realistic environment of the probe touching the brain, the gap size of the SRR is set to 1 × 1 mm and the brain model has contact including the gap area, as shown in Figure 3a. The corresponding SRR sizes in Figure 3b,c are 17.5 × 17.5 and 7 × 7 mm. The resonance of each SRR can be seen in the S-parameter graph in Figure 4. In the simulation with the brain model, it can be seen that the 6.5 GHz SRR shows a S_11_ value of −10 dB, and matching is well performed. In order to make different resonant frequencies with the same capacitance, the SRR size must be varied. The 2.4 GHz SRR occupies a 6.25 times larger area than the 6.5 GHz SRR, and when implemented through a PCB, the 2.4 GHz SRR is 6.25 times heavier. Currently, in the case of a small microscope that can be mounted on the head of a mouse, the overall size is 100 mm^2^ × 22 mm (area × height) [15]. Compared to the conventional mouse head mount device, the size of the 2.4 GHz SRR has not been verified for conformity, whereas the 6.5 GHz SRR is smaller than the verified device size. Considering attenuation, matching to brain tissue, and the size of the probe with a mountable size on the mouse head, it is considered that the 6.5 GHz frequency is suitable for MBS behavioral experiments.

### 2.2. 6.5 GHz Stimulation Probe for Brain Stimulations

**Figure 5:**
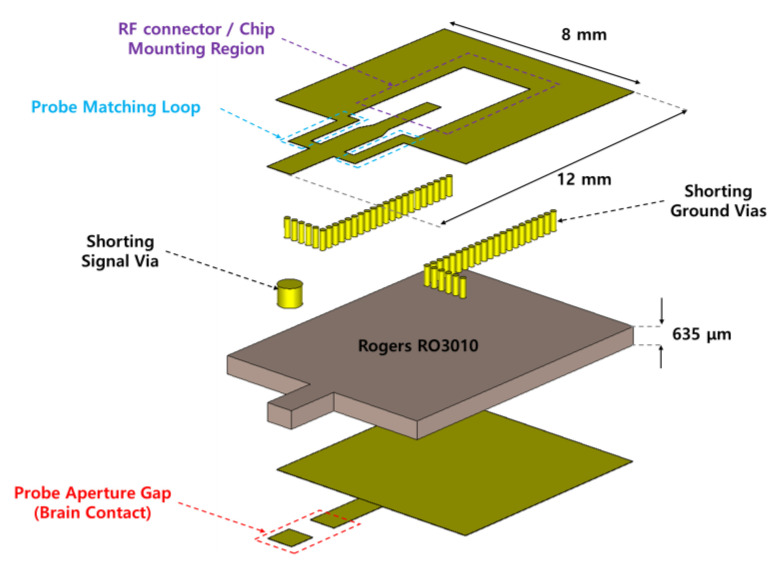
Exploded view of the probe with an RF connector or chip-mounted region and an aperture gap.

**Figure 6:**
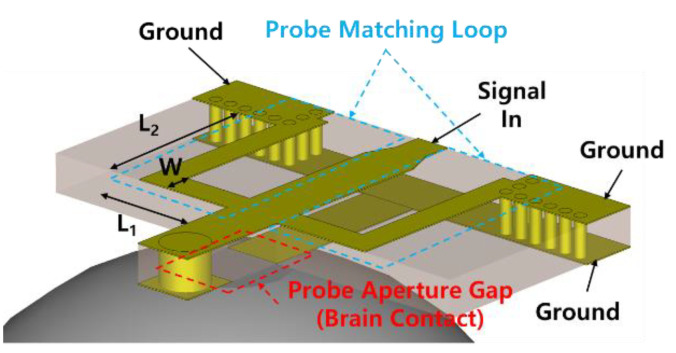
Structure of the proposed probe for E-field focusing and matching to the brain.

This section describes the geometry, frequency response, and the distribution of electric fields within the brain model of the probe for brain stimulations. Figure 5 shows an exploded view of a probe that includes an RF connector or chip-mounted region for implementing a chip-on-probe MBS system and an aperture gap in contact with the brain. The area of the designed probe is 96 mm^2^ or less, which is smaller than the aforementioned 100 mm^2^ reference area. A Rogers RT6010 substrate having a thickness of 635 μm is used as a substrate. It has a dielectric constant and loss tangent of 11.2 and 0.0022, respectively. To deliver the microwave signal to the brain using the 6.5 GHz SRR shown in Figure 3b, it is conventionally fed by inductive coupling through a microstrip line [16]. In this indirect feeding method, there exists some loss by coupling, and the gap between the line and SRR causes the dispersion of the electric field, making it difficult to focus the field onto the desired site. Therefore, in this work, a structure is proposed in which the electric field is formed only in the gap of the SRR via direct feeding to the SRR, as shown in Figure 6. The gap of the SRR is located at the bottom of the PCB, where the brain is in contact, to achieve the intention of delivering the focused electric field to the brain model. When the matching loop of the SRR required for resonance is located on the bottom surface, identically to the gap, the electric field can be distributed by unintentional coupling. Therefore, the loop is moved to the top of the PCB and connected through an electrical via hole to the gap. The signal is inserted into a resonator with low reflection consisting of capacitance formed by a gap and a symmetrical matching loop that appears to be inductance through a grounded coplanar waveguide feed.

**Figure 7:**
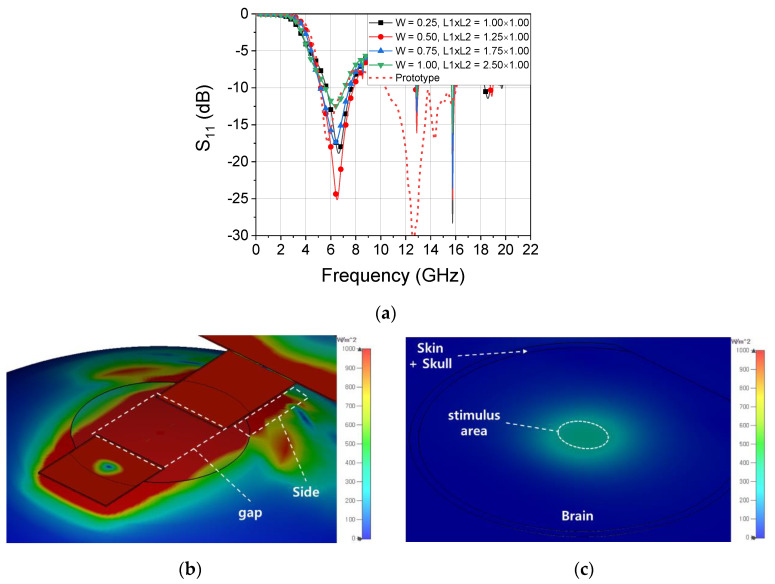
Simulated (**a**) S11 of the probe in contact with the brain for a differently sized matching loop with resonance of 6.5 GHz (**b**) power distribution at the surface of the brain model in contact with the probe and (**c**) within a depth of 3 mm.

Figure 7a plots the S-parameters for cases where the prototype and proposed probe resonates at 6.5 GHz depending on the loop contact location (L2), length (L1 + L2), and width (W) of the matching loop, which is shown in Figure 6. For comparison, the prototype probe has a structure in which the chip is mounted directly to the probe PCB without an auxiliary PCB. Similar to the proposed probe with various dimensions, the prototype also has a return loss of 12 dB at 6.5 GHz, which is matched to 50 Ohms. As the capacitance value is already fixed in the proposed structure, the inductance value required for 6.5 GHz resonance is determined. As *W* increases, the length of the loop required to form an identical amount of inductance increases. During the simulation, the average increase in the length should be 0.5 mm for every increase in *W* of 0.25 mm. Table 1 summarizes the geometry, the insertion loss (IL) at 6.5 GHz, and the 10 dB impedance bandwidth of the proposed probe including the aforementioned modified SRR structure. A total of 12 different modified SRR geometries are used with the probe to acquire resonance at 6.5 GHz. The dimensions of the loop with the best impedance matching to the brain model are a width of 0.5 mm and a 1.25 × 1.0 mm loop size, where IL shows −25.1 dB at 6.5 GHz and a 40.2% of the impedance bandwidth. Figure 7b,c presents simulation results using CST Studio, showing the distribution of the electric field formed at the surface and 3 mm deep when a 10 dBm stimulus signal is applied by attaching a probe to the brain, respectively. In the simulation, a more realistic environment can be utilized through a brain model including the skin and skull. The RF power on the surface of the brain model is distributed not only in the gap of the probe but also on the side of the line. It can be seen in Figure 7c that the power distributed over such a large surface is limited only to the skin surface and is highly concentrated in terms of the distribution at a depth of 3 mm in the model. The targeted hippocampal CA1 neurons are located at 1.9 mm ventral, 1.4 mm lateral to bregma, and 1.2 mm depth from the surface. The field distribution was analyzed at about 3 mm from the probe surface considering the location of the target at 1.2 mm deep from the brain surface and the average thickness of the scalp and skull [17,18]. In a brain model with a 3 mm depth, the stimulus area where power is distributed over 645 W/m^2^ or more has an oval shape with a long diameter of 2.7 mm and a short diameter of 2 mm [19].

### 2.3. Chip-on-Probe Design: Suppressed Heat Transfer to the Brain

**Figure 8:**
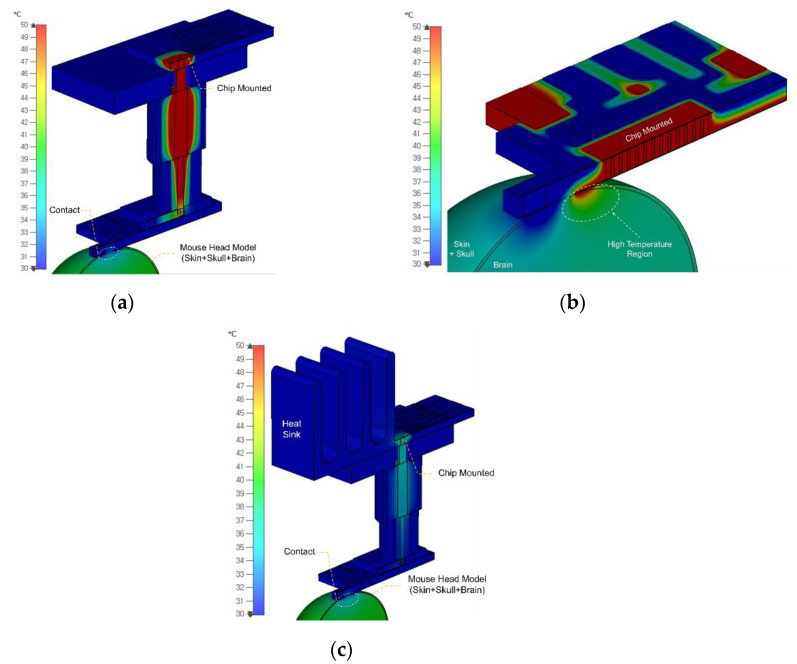
Simulated (**a**) direct chip-on-probe, (**b**) chip-on-probe using an auxiliary PCB with a RF connector, and (**c**) heat sink added for 2 W chip heat dissipation.

**Figure 9:**
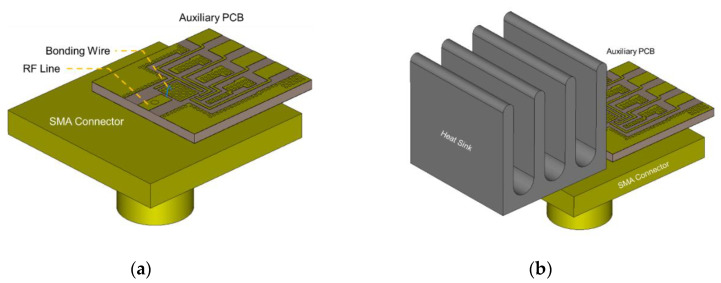
Transition structure of the auxiliary PCB and SMA connector (**a**) without and (**b**) with a heat sink.

In order to remove the RF cable required for mouse behavioral experiments, the design of a chip-on-probe topology is intended. Generally, the temperature that does not damage the brain of the mouse is known to be below 39 °C [20]. When the chip is mounted directly on the probe, heat from the chip is delivered directly through the probe’s electrical vias to the brain, which can cause temperature problems. Figure 8a shows a temperature distribution using CST MPHYSICS Studio of how much heat generated by a chip consuming 2 W DC power causes a temperature change in the brain when the direct chip-on-probe comes into contact with the brain model. The power consumption of the chip used in the chip-on-probe system is calculated based on a laboratory-made 6.5 GHz stimulus-signal-generating chip using a conventional cross-coupled voltage-controlled oscillator and a two-stage differential power amplifier topology. The area marked as the high-temperature region in Figure 8a is where the brain’s internal temperature exceeds 50 °C, which affects a brain with a depth of 1 mm or more. To avoid this temperature problem, this paper proposes a new topology using an auxiliary PCB to mount the chip. The auxiliary PCB has a square pattern on which the chip can be mounted and pads with lines to supply DC voltage to the chip. Additionally, an RF line exists for the connection between the attached SMA connector and the chip RF output pad with a hole for inserting the signal pin of the connector, as shown in Figure 9. The auxiliary PCB increases the temperature stability of the entire system by not only physically increasing the distance between the brain and the heat-generating chip but also securing an additional heat-sink mounting space. As shown in Figure 7b, most of the heat is distributed only to the auxiliary PCB, where the chip is attached to it and the connector.

The temperature formed inside the brain is 39 °C or less, indicating that there is no temperature effect. If the additionally secured heat sink is attached to the SMA connector and auxiliary PCB, the temperature at the probe aperture can be lowered by more than 2 °C by effectively releasing the heat of the chip, as shown in Figure 8c. The designed heat sink is 1 × 1 × 1 cm^3^ in size, has four heat-sinking bars with a thickness of 1 mm, and is attached through a heating pad. Considering the application to be mounted on a mouse, instead of copper with good thermal conductivity, aluminum, which has a specific gravity of 2.7 (one-third that of copper), was selected. Therefore, it was confirmed in a simulation that the heat generated by the chip transferred to the brain was effectively suppressed without affecting the brain tissue using the vertically arranged auxiliary PCB and the attached heat sink.

### 2.4. Design of the Transition Structure between the Probe Aperture and Auxiliary PCB

A low-loss transition structure is required to deliver the signal from the chip’s output pad to the end of the SMA connector. As the proposed 6.5 GHz probe was designed considering that a SMP connector would be attached, the transmission loss caused by the vertically attached auxiliary PCB and connector was simulated. Figure 9 presents the layout of the transition structure of the SMA connector from the chip mounted on the auxiliary PCB without and with a heat sink.

**Figure 10:**
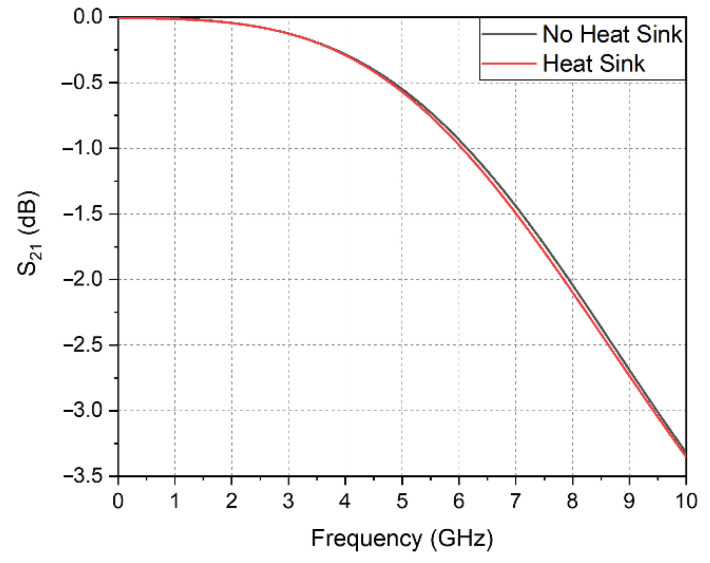
Simulated S21 of the transition probe in contact with the brain for differently sized matching loops with 6.5 GHz resonance.

The transferred loss from the chip’s output pad to the end of the SMA connector is shown in Figure 10. The loss of the simulated transition structure at 6.5 GHz is 1.1 dB, including the wire bonding effect. Given that the designed aluminum heat sink is attached over the SMA connector near the RF line, an effect of the heat sink on the RF signal is seen. As shown in Figure 10, even when the heat sink is attached, the loss in the transition structure is 1.2 dB, i.e., only a 0.1 dB difference. Through this, it was confirmed that the heat dissipation of the chip that occurs during the in-vivo experiment is solved by the heat sink. Thus, this design is feasible for use as it also has a negligible effect on the RF performance.

## 3. Fabrication and Measurement

**Figure 11:**
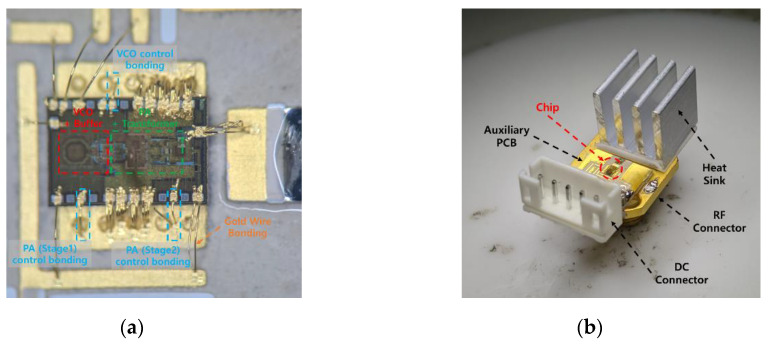

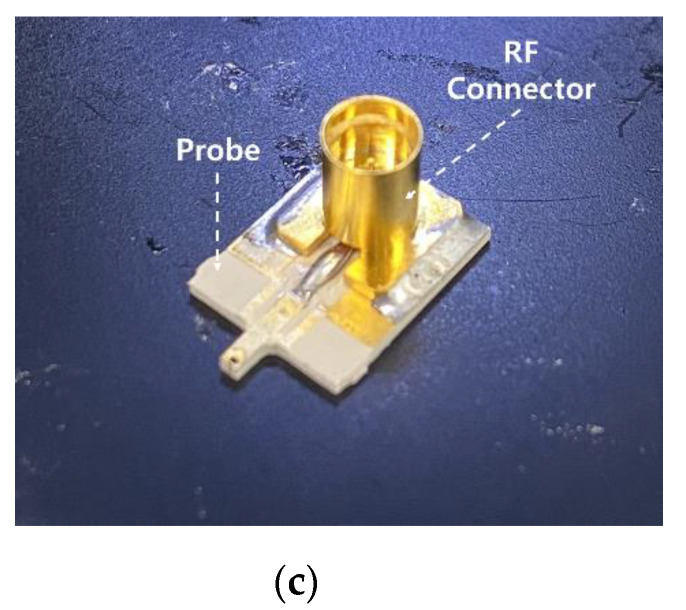
Photograph of the fabricated (**a**) chip, (**b**) auxiliary PCB with supplementary parts, and (**c**) probe.

Figure 11a shows a top view of the fabricated chip mounted on an auxiliary PCB. VCO, a buffer, the PA, and the transformer circuits are all integrated on a single chip and fabricated in the 0.28 μm silicon-on-insulator CMOS process. Each pad of the chip is connected to a ground or a metal line with DC voltage applied through gold wire bonding. Three grounded wire bondings were added to control the oscillation condition and the gate voltage of each stage’s common-source amplifier of the PA. Figure 11b shows a photograph of the manufactured auxiliary PCB with the supplementary parts of the chip, RF connector, DC connector, and heat sink. Appropriate voltages are applied through the DC connector to drive the chip, and its performance can be verified by connecting measurement equipment to the RF connector. The manufactured probe is connected to a 90°-angled RF connector to receive stimulus signals from the chip.

The fabricated chip is driven with a 3.4 V drain voltage and consumes DC current of 650 mA in the on state and 71 mA in the off state during modulation. The frequency generated by the chip can be tuned between 5.7 and 7.1 GHz. With the modulation switch turned off, the output power varies from −1 to 11.4 dBm at 6.5 GHz. Rectangular pulse modulation of the stimulus signal is utilized by turning the gate of the PA and VCO current source transistor on and off.

**Figure 12:**
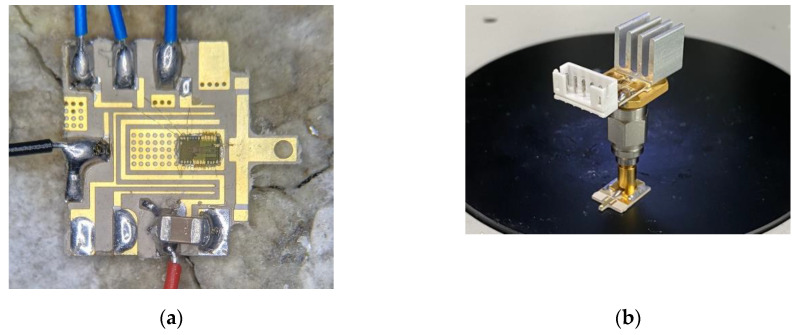

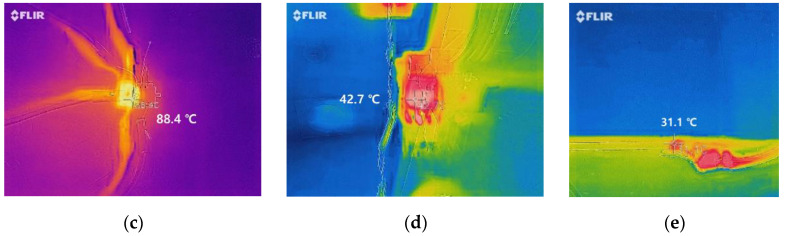
Photograph of the fabricated chip-on-probe (**a**) without and (**b**) with auxiliary PCB, a thermal image with chip temperature under the operation of (**c**) without and (**d**) with auxiliary PCB, (**e**) temperature at the probe bottom surface where mouse brain is contact.

A thermal imaging camera (FLIR ONE pro) was used to compare the heat generation of the chip when the chip was directly mounted on the probe and when a physical distance existed with the RF connector using the auxiliary PCB. Figure 12a is a photograph of a directly connected chip-on-probe fabricated for a thermal comparison of the proposed system, as shown in Figure 12b. Figure 12c,d depicts thermal images of the maximum temperature of the chip when both the directly connected and the proposed chip-on-probe systems are operated at the same DC bias voltages. While the chip is in operation at an output power of 11 dBm, the directly connected topology has a maximum temperature of 88.4 °C, whereas the topology where the physical distance exists between the chip and the probe using the auxiliary PCB shows a temperature of 42.7 °C. As shown in Figure 12e, the heat generated from the chip is evenly dissipated through the auxiliary PCB, heat sink, and RF connector, which has a cooling effect of 45.7 °C. In an animal experiment, the temperature of the bottom of the probe, which is critical due to its direct attachment to the brain tissue, was 31.1 °C, and it was confirmed to prevent side effects that may be caused by heat.

## 4. Conclusions

This paper proposed a novel design approach to suppress the heat transfer of a chip to biological tissue in a system in which a probe and a chip are combined without using the RF cable. The proposed approach was validated with a probe that allows the matching of brain tissue with the chip and a chip that generates, modulates, and amplifies signals at a microwave frequency of 6.5 GHz. The frequency of 6.5 GHz was chosen from a theoretical study that considers the trade-off between the attenuation of the field inside the brain tissue and the probe size. In order to suppress the temperature transfer to the brain, instead of mounting the chip directly on the probe, it was mounted onto an auxiliary PCB, with the PCB connected through the probe with an RF connector. The loss in the transition path from the probe aperture surface to the pad of the chip was designed and simulated and the proposed system was fabricated. The total combined weight of the probe, chip, auxiliary PCB, heat sink, DC connector, and the RF connector is 2.7 g, which has a 1.3 g margin compared to the 4 g weight limit. Compared to the directly attached system, a chip temperature of 42.7 °C with a reduction of 45.7 °C was measured, and the temperature of the probe aperture surface in contact with the brain was 31.1 °C when operating the chip with an output power of 11 dBm. For the first time, the MBS system using the proposed topology does not cause any temperature problems on brain tissue within the weight limit that enables behavioral tests with a mouse model.

## Figures and Tables

**Table 1:** Geometric parameters, insertion losses, and impedance bandwidths of the modified SRR for 6.5 GHz brain-to-probe matching.

W (mm)	L_1_ (mm)	L_2_ (mm)	Total Length = L_1_ + L_2_ (mm)	Insertion Loss(dB)	Impedance Bandwidth (GHz)
0.25	1.00	1.00	2.00	18.5	5.64–7.62 (29.9%)
0.25	1.00	1.50	2.50	18.3	5.42–7.52 (32.5%)
0.25	1.00	1.75	2.75	15.4	5.72–7.28 (24.0%)
0.50	1.00	0.50	1.50	16.0	5.72–8.02 (33.5%)
0.50	1.25	1.00	2.25	25.1	5.2–7.82 (40.2%)
0.50	1.50	1.25	2.75	18.3	5.44–7.56 (32.6%)
0.75	1.75	1.00	2.75	17.1	5.18–7.5 (36.6%)
0.75	2.00	1.00	3.00	16.8	5.4–7.6 (33.8%)
0.75	2.25	1.00	3.25	16.0	5.6–7.66 (31.1%)
1.00	2.50	1.00	3.50	12.4	5.56–7.1 (24.3%)
1.00	2.75	1.00	3.75	12.2	5.76–7.18 (21.9%)
1.00	3.00	1.00	4.00	12.0	5.92–7.24 (20.1%)
